# Supplementary material for: Sigma-1 receptor chaperones rescue nucleocytoplasmic transport deficit seen in cellular and Drosophila ALS/FTD models
Source: Nat Commun. 2020 Nov 4;11:5580. doi: 10.1038/s41467-020-19396-3 (PMC7642387; doi:10.1038/s41467-020-19396-3)
Supplement: Supplementary file 1 — Supplementary Information [file 41467_2020_19396_MOESM1_ESM.pdf]

SUPPLEMENTARY INFORMATION

Sigma-1 receptor chaperones rescue nucleocytoplasmic transport deficit seen in cellular and *Drosophila* ALS/FTD models

Lee et al.

1)Supplementary Figures

2)Supplementary Tables (Antibodies and Reagents)

3)Original Western Blot PVDF Membranes (including those in the main text and supplementary).

NOTE: For detection of multiple proteins in one PVDF membrane, the segment of protein of interest was usually cut per guide of color M.W. markers and then individually blotted with respective antibody. The reasons are two-fold: to save antibodies and to avoid protein loss that would have occurred if reblotting the whole membrane by repetitive stripping.

Supplementary Fig. S1

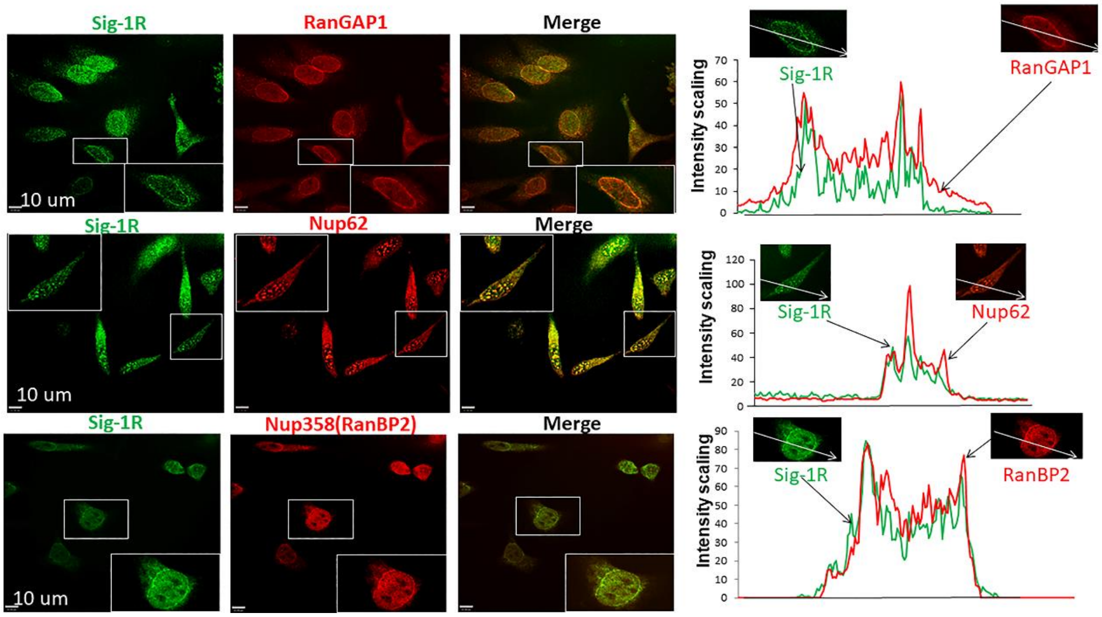

Supplementary Fig.S1. Immunocytochemical colocalization of endogenous Sig-1R with RanGAP1, Nup62, and RanBP2 in HeLa cells. Endogenous Sig-1R was labeled with B5 monoclonal antibody (Santa Cruz Cat#SC-137075). Right panels show the tracing of signal intensity as the arrow traverses the indicated portion of a cell. The experiment was independently examined three times using biologically independent samples with similar results.

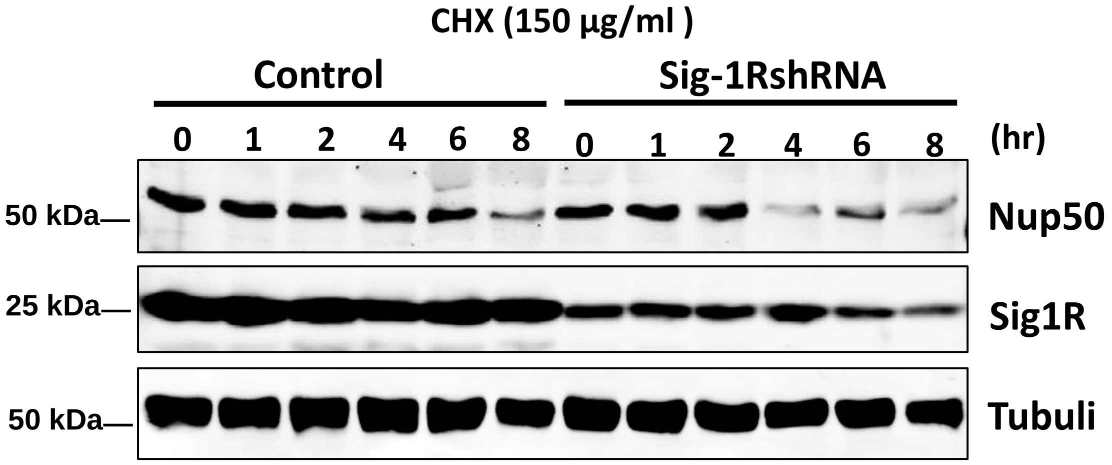

Supplementary Fig.S2. Turnover of NuP50 in wild type or Sig-1R knockdown HeLa cells. Cycloheximide (CHX; 150 µg/ml) was added to stop *de novo* synthesis of proteins. Time-lapsed levels of NuP50 were examined by western blot probed by the antiNuP50 antibody. The experiment was independently examined three times using biologically independent samples with similar results.

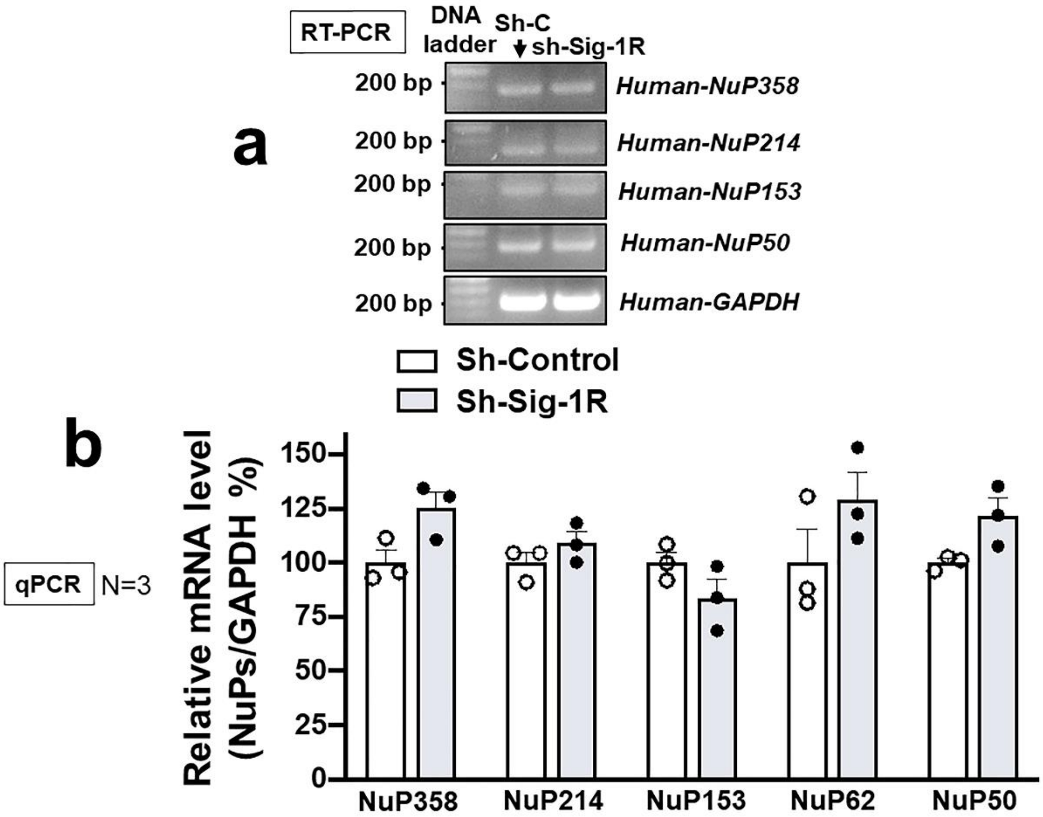

Supplementary Fig.S3. RT-PCR and quantitative real-time PCR of NuPs in wild type or Sig-1R knockdown HeLa cells. Total RNAs were extracted from wild type or transfected cells. The mRNA levels of NuP 358, NuP214, NuP153, NuP50 and GAPDH were detected by RT-PCR (upper panel). The quantitative real-time PCR (qPCR) was performed to measure mRNA levels of NuPs by using specific primers for amplifications (lower panel). The mRNA expression level of NuPs were normalized to house-keeping gene *GAPDH*. The experiment was independently examined three times using biologically independent samples with similar results. Data presented are means ± SEM.

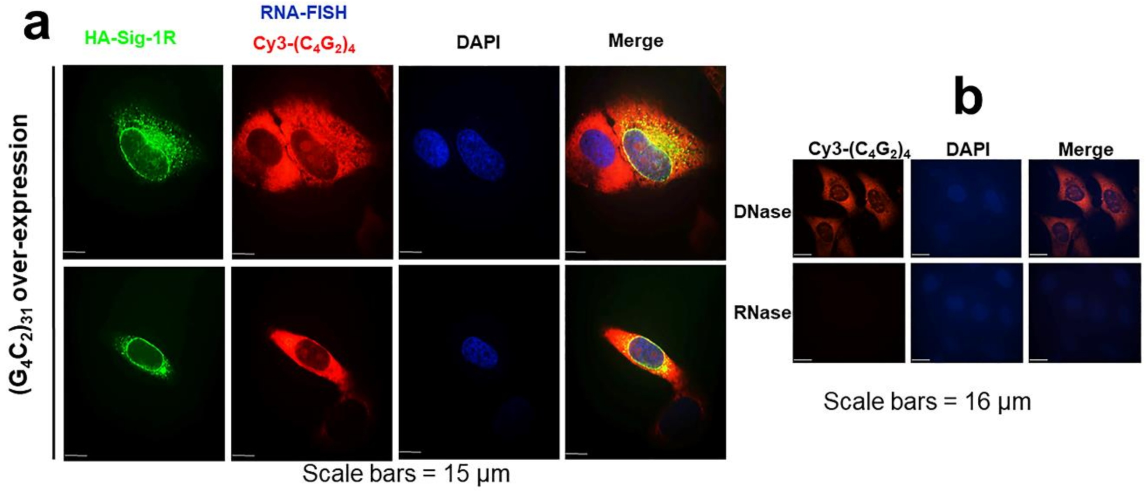

Supplementary Fig.S4. Sig-1R colocalizes with (G4C2)31-RNA at the perinuclear area. (a) HeLa cells were transiently co-transfected with $(G_4C_2)_{31}$/pcDNA5 and HA-Sig-1R vectors. Cells were then fixed and hybridized with Cy3-$(C_4G_2)_4$ (red fluorescence) in the RNA fluorescent *in situ* hybridization (FISH) assay. Cells were then stained with HA antibody for detection of HA-Sig-1R (green fluorescence). DAPI, nucleus. The image data were visualized by confocal microscope. **(b)** Verification of the RNA products from $(G_4C_2)_{31}$/pcDNA5 transfection by using NG-108 cells. Cells were transiently transfected with $(G_4C_2)_{31}$/pcDNA5 in the presence of DNase or RNase, then cells were fixed and hybridized with Cy3-$(C_4G_2)_4$ to perform RNA fluorescence *in situ* hybridization (red fluorescence). The results were imaged by confocal microscopy. Nuclear structure was visualized by DAPI staining (blue). The experiment a was independently examined three times using biologically independent cells with similar result. The experiment b was independently examined two times using biologically independent cells with similar results.

Supplementary Fig. S5

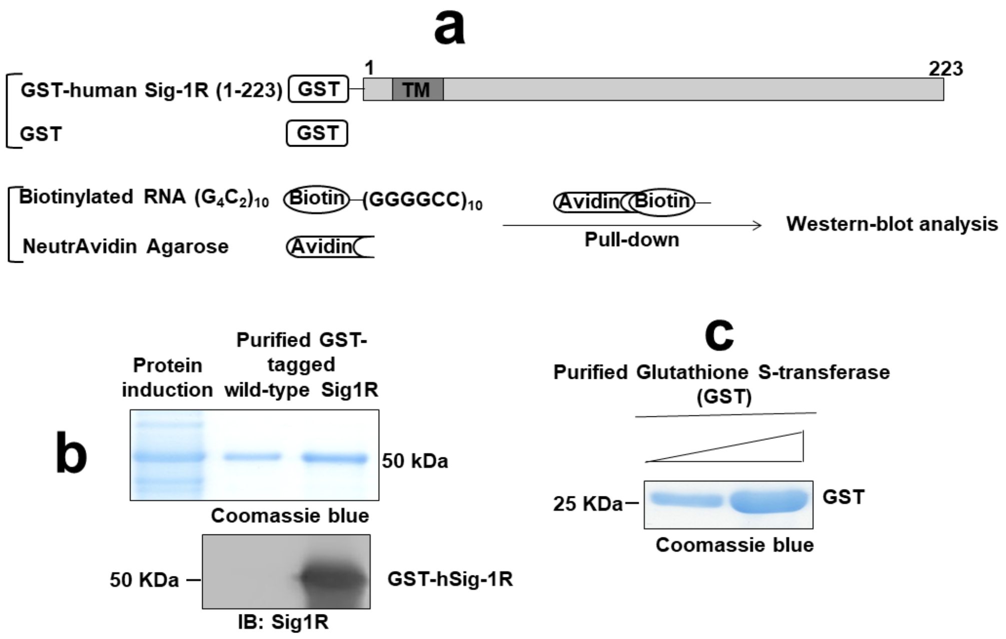

Supplementary Fig.S5. Illustration of GST-Sig-1R and its use in the biotin pull down assay. **(A)** Illustration of full-length GST-tagged recombinant human Sig-1R and the principle of the biotin pull down assay are shown. **(B)** The GST-tagged recombinant Sig-1R protein and its identity probed with the Sig-1R antibody in western blot. **(C)** The control protein GST alone was purified from *E. coli.* and is shown with coomassie blue. The experiments b and c were independently examined two times using biologically independent samples with similar results.

Supplementary Tables

Table S1: Antibodies

Note: Each commercially available antibody has been tested by each respective company and was used at the recommended dilution ratio or adjusted experimentally. For the in-house customed-made Sig-1R antibody (#5460), its specificity was demonstrated in the article published from the authors' laboratory (Hayashi and Su, Cell, 131: 596-610).

**Antibodies**	**Dilution ratio<br>W: western blot<br>I: immunostaining**	**Antibody companies**	**Product code**
Rabbit polyclonal anti-Ran	W: (1:1000)<br>I: (1:200)	Abcam	Ab53775
Rabbit polyclonal anti-RanGAP1	W: (1:1000)<br>I: (1:200)	Cell Signaling Technology	#36067
Rabbit polyclonal anti-RanBP2 (i.e., NuP358)	I: (1:200)	Abcam	Ab64276
Mouse monoclonal anti-mab414 (recognizing FG-repeat-containing NuP62, NuP214, NuP358)	W: (1:1000)<br>I: (1:200)	Abcam	Ab24609
Rabbit polyclonal anti-Nup50	W: (1:1000)	Abcam	Ab137029
Mouse monoclonal anti-Nup62	W: (1:1000)	BD Biosciences	610498
Rabbit polyclonal anti-GST	W: (1:1000)	Cell Signaling Technology	#2622
Normal Rabbit IgG	NA	Cell Signaling Technology	#2729
Normal Mouse IgG	NA	Santa Cruz Biotechnology	sc-2762
Mouse monoclonal anti-Sigma-1 receptor	W: (1:1000)	Santa Cruz Biotechnology	sc-137075 (a.k.a. B5)

Rabbit polyclonal anti-guinea pig Sigma-1 receptor (a.a. 144-165) (Hayashi and Su, 2003)	W: (1:1000)	In-House (Custom-made by QCB)	#5460
Rabbit plyclonal anti-human Sigma-1 receptor (a.a. 142-161)	W:(1:100)	In-House (Custom-made by Abliance, Campiegne, France)	No catalog number
Rat anti-Elav antibody	W:(1:700)	Developmental Studies Hybridoma Bank, Iowa City, Iowa	7E8A10
Mouse monoclonal anti-HA	W: (1:1000)	Santa Cruz Biotechnology	sc-7392
Rabbit polyclonal anti-Sp1	W: (1:1000)	Santa Cruz Biotechnology	sc-59
Mouse monoclonal anti-RanBP-2	I: (1:200)	Santa Cruz Biotechnology	sc-74518
Mouse monoclonal anti-V5	W: (1:1000)	Thermo Fisher Scientific	R962-25
Mouse monoclonal anti-alpha-Tubulin	W: (1:20,000)	Sigma-Aldrich	T5168
Mouse monoclonal anti-β-actin	W: (1:5,000)	Sigma-Aldrich	A5441
Rabbit polyclonal anti-HA	W: (1:1000)	Sigma-Aldrich	H6098
Rabbit polyclonal anti-GFP	W: (1:1000)	GeneTex	GTX113617
IRDye 800CW Goat anti-Mouse IgG (H+L)	W: (1:1000)	LI-COR Biosciences	925-32210
IRDye 680RD Goat anti-Rabbit IgG (H+L)	W: (1:1000)	LI-COR Biosciences	925-68071
Alexa Fluor 488 Goat anti-Mouse IgG (H+L)	I: (1:200)	Thermo Fisher Scientific	A11092
Alexa Fluor 488 Goat anti-Rabbit IgG (H+L)	I: (1:200)	Thermo Fisher Scientific	A11034
Alexa Fluor 594 Goat anti-Mouse IgG (H+L)	I: (1:200)	Thermo Fisher Scientific	A11032
Alexa Fluor 594 Goat anti-Rabbit IgG (H+L)	I: (1:200)	Thermo Fisher Scientific	A11037

Peroxidase-conjugated affinipure goat anti-mouse IgG	W: (1:20,000)	Jackson ImmunoResearch Lab.	115-035-164
Peroxidase-conjugated IgG fraction monoclonal mouse anti-rabbit IgG, light-chain	W: (1:20,000)	Jackson ImmunoResearch Lab.	211-032-171
**Supplies:**			
Protein A magnetic beads		Millipore	LSKMAGA02
Protein G magnetic beads		Millipore	LSKMAGG10
Glutathione Sepharose 4B		GE Healthcare	17-0756-01
NutrAvidin Agarose Resin		Thermo Fisher Scientific	29201
Cy3-(C4G2)4 for RNA FISH		Integrated DNA Technologies	Custom made
Subcellular Protein Fractionation Kit for Cultured Cells		Thermo Fisher Scientific	78840

Table S2: Primer pairs for vectors and nucleotide sequence for biotin-labeled RNA

**Primer pairs used for vector constructions**				
**CDS of Gene**	**Accession Number**	**Primer pairs (5′ to 3′)**	**RE sites**	**amplicon size (bp)**
**Human Sig-1R**	NM_005866	**S: GGAATTCCCAGTGGGCCGTGGGCCGGCGGTGG**<br>**AS: CCGCTCGAGCGGAGGGTCCTGGCCAAAGAGGTAGGT**	**EcoRI**<br>**XhoI**	
**Human Sig-1R-E102Q**	NM_005866	**S: CACGCCTCGCTGTCCCAGTATGTGCTGCTCTT**<br>**AS: AAGAGCAGCACATACTGGGACAGCGAGGCGTG**	**EcoRI**<br>**XhoI**	
**Mouse Sig-1R**	NM_011014	**S: GGAATTCCCCGTGGGCCGCGGGACGGCGGTGG**<br>**AS: CCGCTCGAGCGGGGAGTCTTGGCCAAAGAGGTAGGT**	**EcoRI**<br>**XhoI**	
**Primer pairs used for quantitative real-time PCR**				
**mRNA of Gene**	**Accession Number**	**Primer pairs (5′ to 3′)**	**Amplicon size (bp)**	
**Human Nup358**	**L41840**	**S: CACCCCAATTTCAAGGGATT**<br>**AS: CGGGCATTTGAACTACTGGT**	**169**	

Human Nup214	NM_005085	S: CACCCCAATTTCAAGGGATT<br>AS: CGGGCATTTGAACTACTGGT	123
Human Nup153	NM_001278209	S: ACCAGTGATAGGCACTTGGG<br>AS: ACAAGTTCCAGGTTTCGGTG	100
Human Nup62	NM_153719	S: GAGTCAACGGATTTGGTCGT<br>AS: GACAAGCTTCCCGTTCTCAG	150
Human Nup50	NM_007172	S: AACATGGCCAAAAGAAATGC<br>AS: AAGGCTCCTCCAGTGTCAGA	176
Human GAPDH	NM_002046	S: GAGTCAACGGATTTGGTCGT<br>AS: GACAAGCTTCCCGTTCTCAG	185
Drosophila Actin	NM_167053	forward 5′- GCGCGGTTACTCTTTCACCA-3′ and reverse 5′- ATGTCACGGACGATTTCACG-3′	51
(G4C2)30	Not applicable	forward 5′-GGGATCTAGCCACCATGGAG-3′ and reverse 5′- TACCGTCGACTGCAGAGATTC-3′	Not applicable

Biotin-labeled RNAs for pull-down assay (Custom-made by Integrated DNA Technologies)

Biotin-labeled RNAs	Nucleotide sequence
Biotin-$(G_4C_2)_{10}$	GGGGCCGGGGCCGGGGCCGGGGCCGGGGCCGGGGCCGGGGCCGGGGCCGGGGCCGGGGCC
Biotin-$(A_2U_2GC)_{10}$	AAUUGCAAUUGCAAUUGCAAUUGCAAUUGCAAUUGCAAUUGCAAUUGCAAUUGCAAUUGC

All Original Western Blots as follows.

Fig.1b

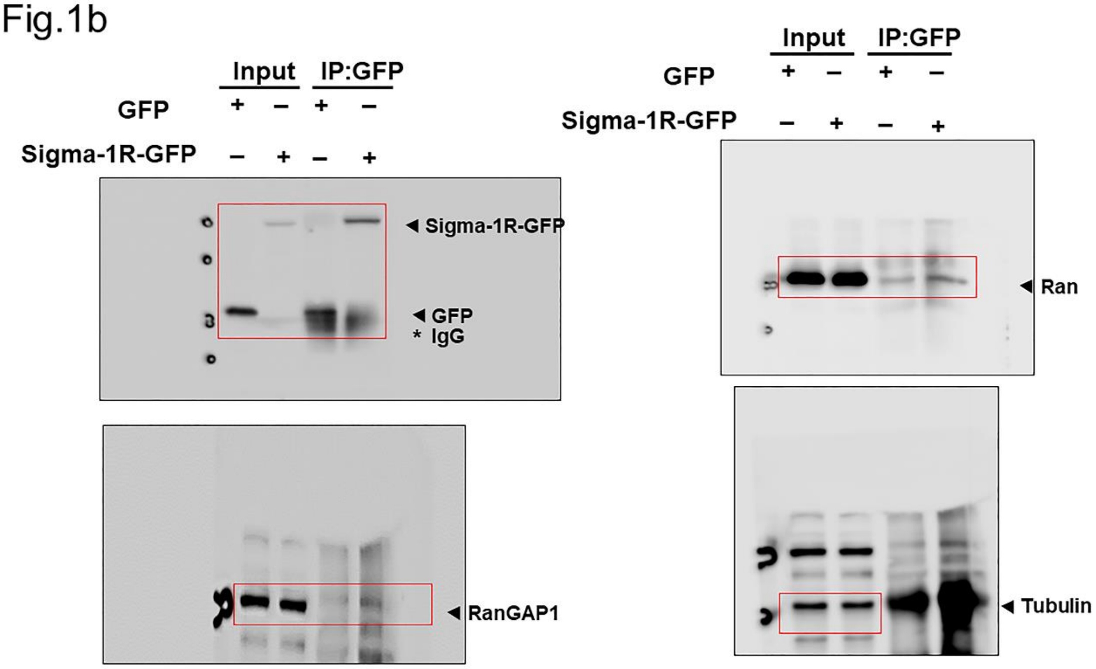

Fig.1c

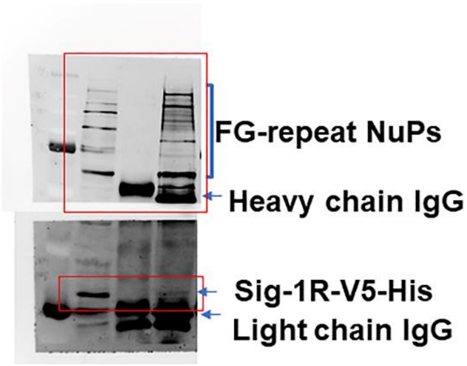
FG-repeat NuPs
Heavy chain IgG
Sig-1R-V5-His
Light chain IgG

Fig.1d

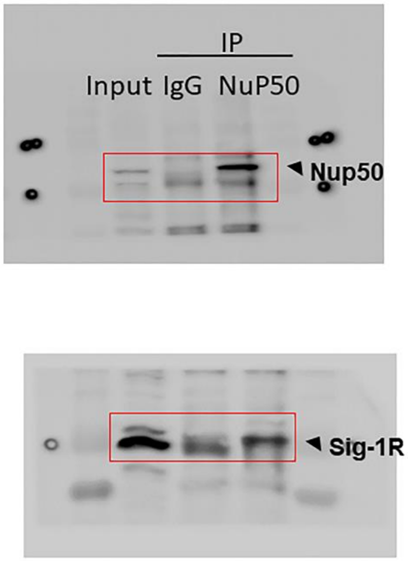
IP
Input IgG NuP50
Nup50
Sig-1R

Fig. 3a

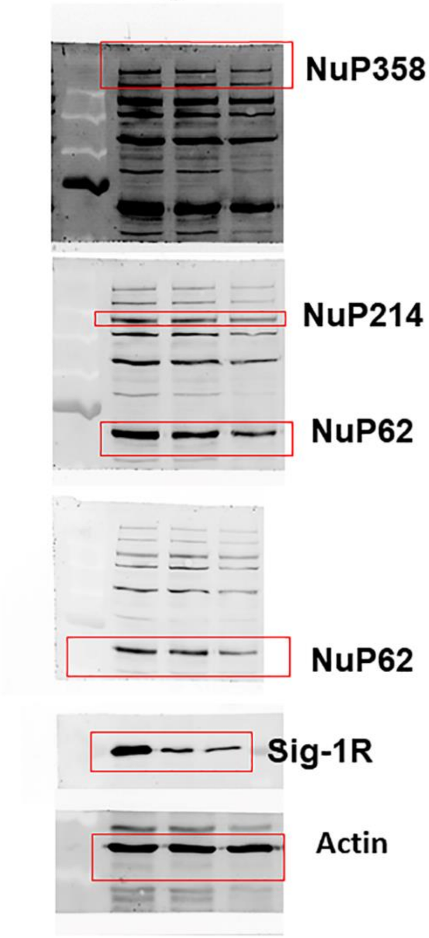

Fig. 3b

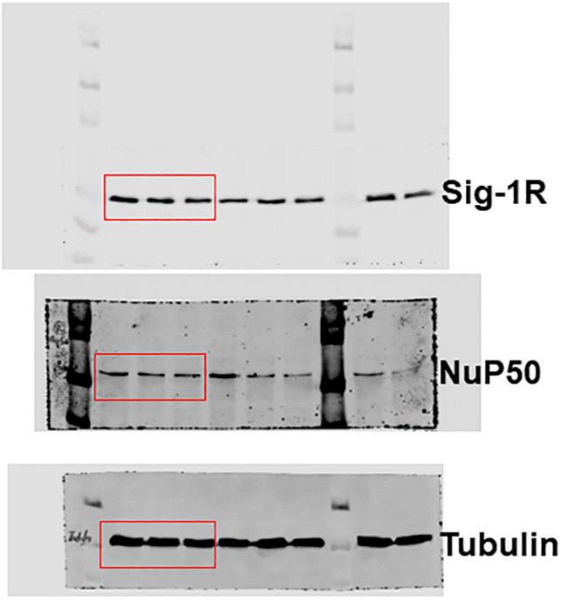

Fig. 3c (three independent experiments)

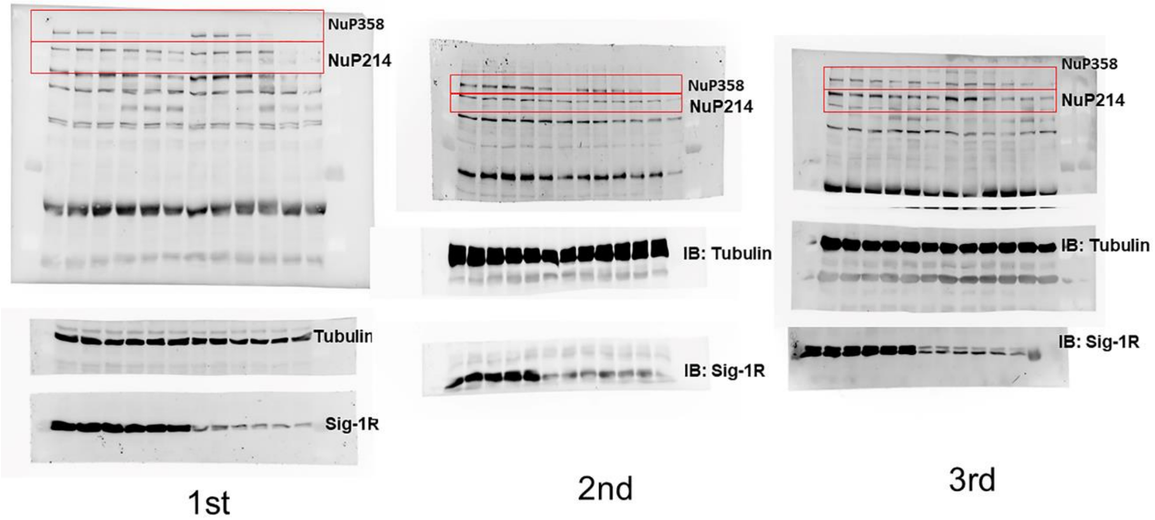

Fig. 4a

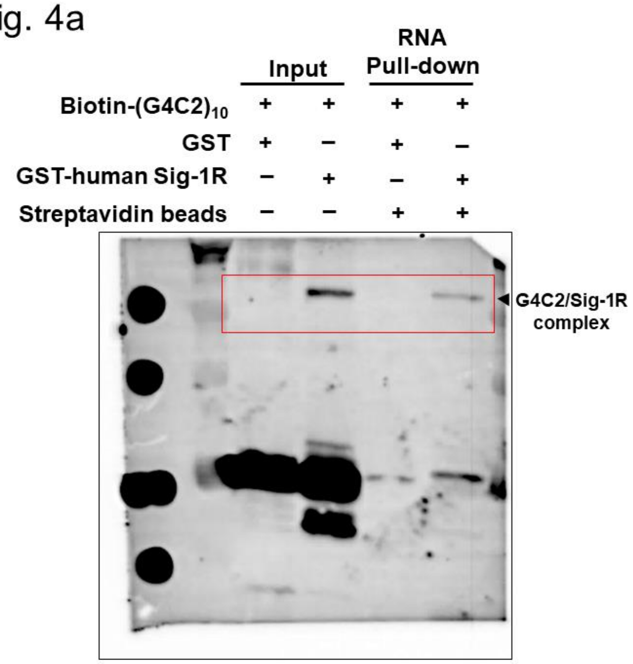

Fig. 4b

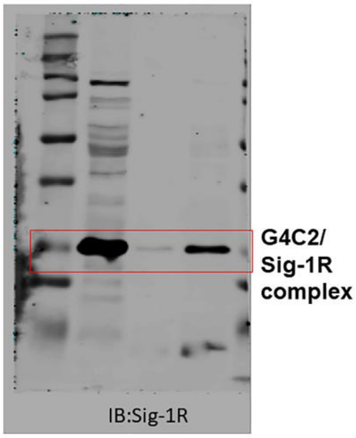

Fig. 4c

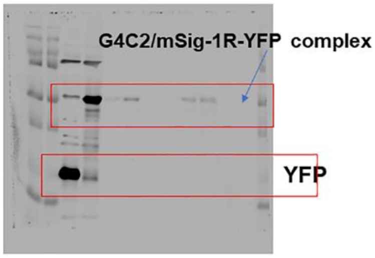

Fig. 4d

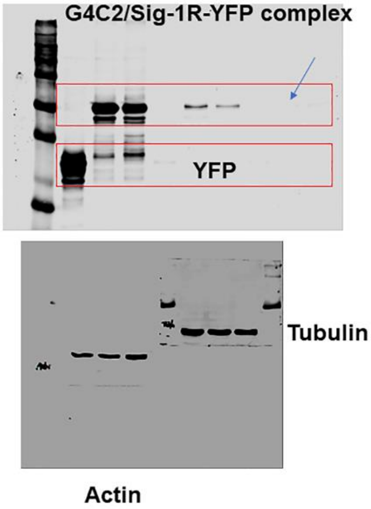

Fig. 6c

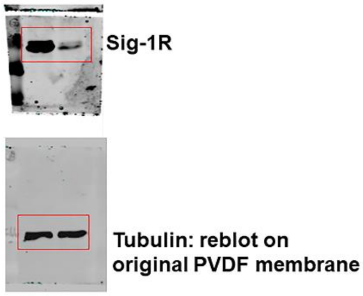

Fig. 7a (three independent experiments)

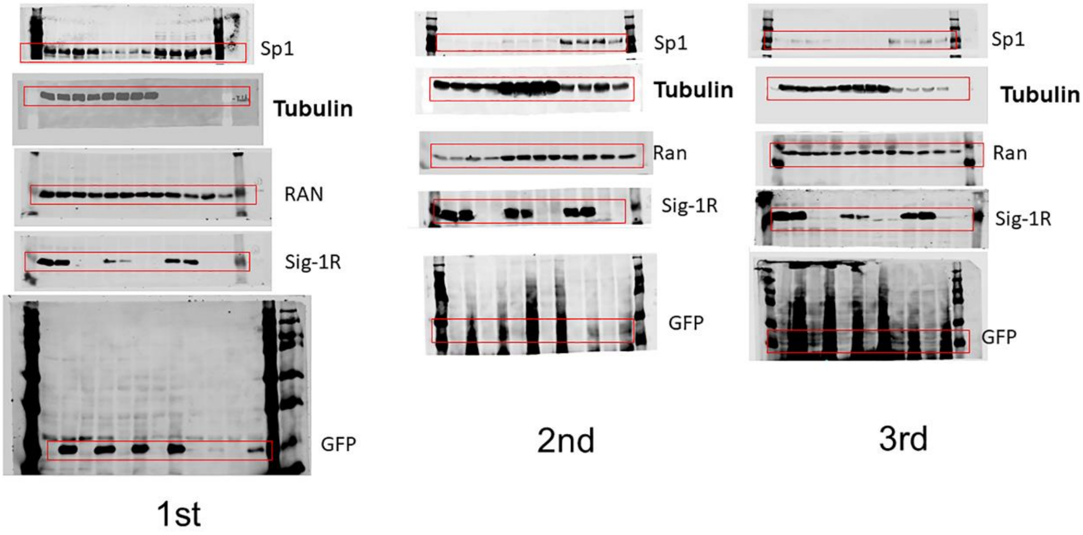

Fig. 7b

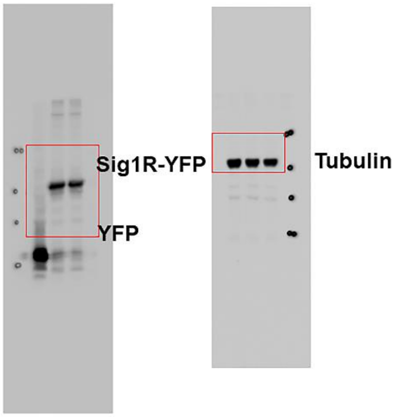

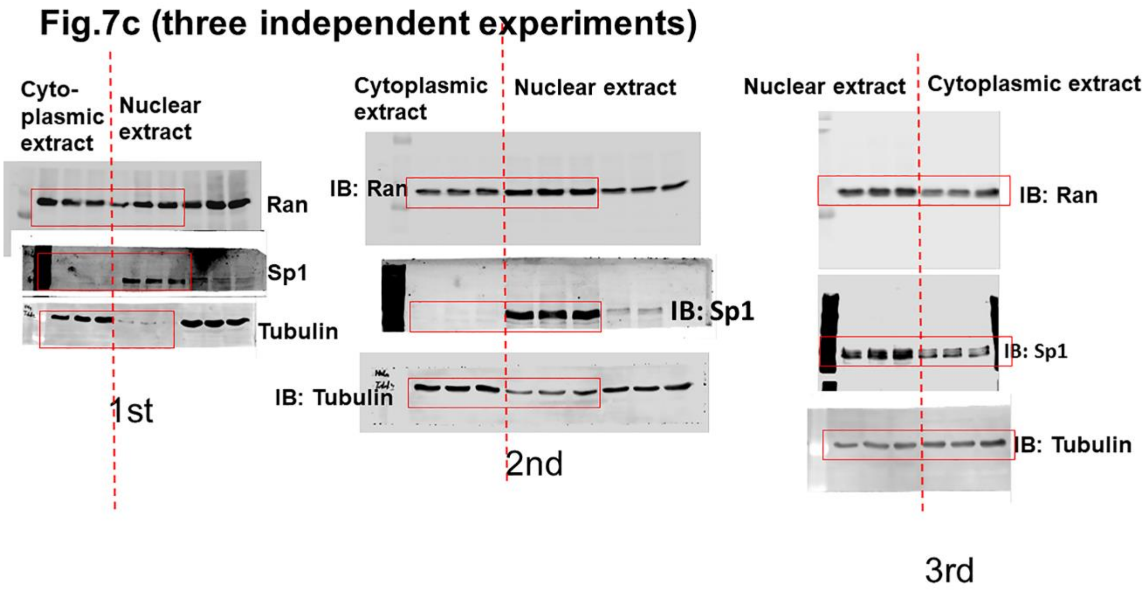
Fig.7c (three independent experiments)
Cyto-plasmic extract
Nuclear extract
Ran
Sp1
Tubulin
1st
Cytoplasmic extract
Nuclear extract
IB: Ran
IB: Sp1
IB: Tubulin
2nd
Nuclear extract
Cytoplasmic extract
IB: Ran
IB: Sp1
IB: Tubulin
3rd

Fig. 8a

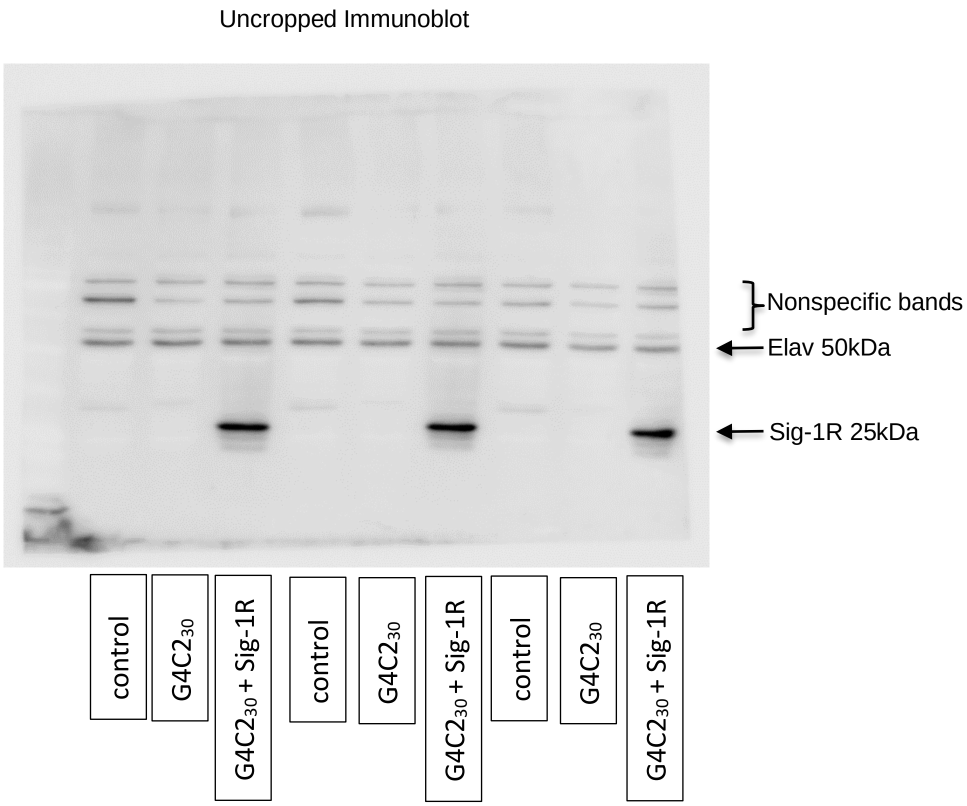
Uncropped Immunoblot
Nonspecific bands
Elav 50kDa
Sig-1R 25kDa
control
G4C2₃₀
G4C2₃₀ + Sig-1R
control
G4C2₃₀
G4C2₃₀ + Sig-1R
control
G4C2₃₀
G4C2₃₀ + Sig-1R

Original western blots for Supplementary Figures as follows.

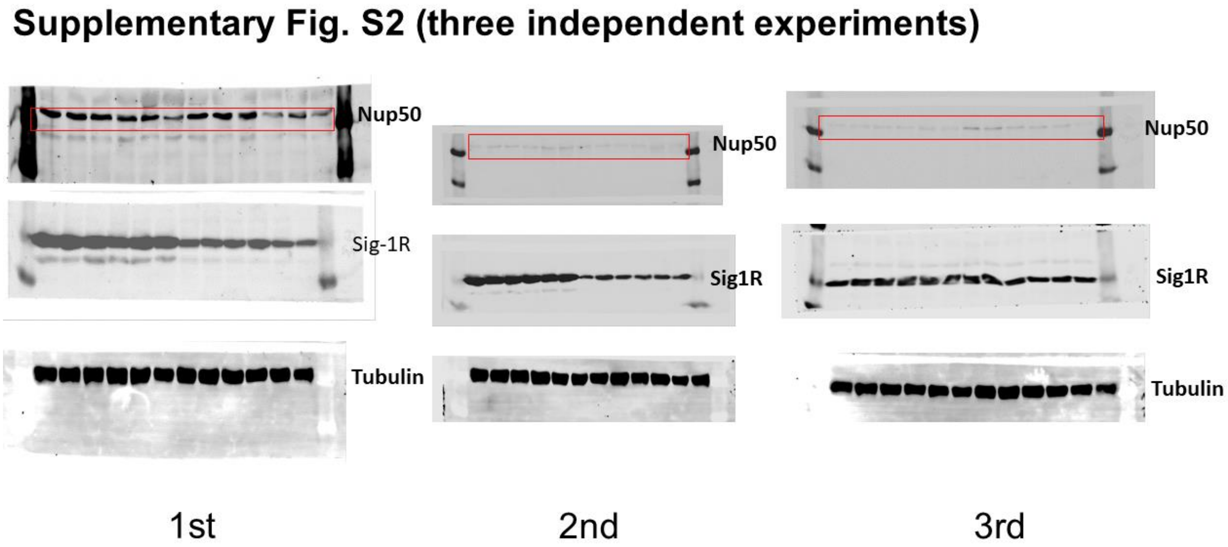

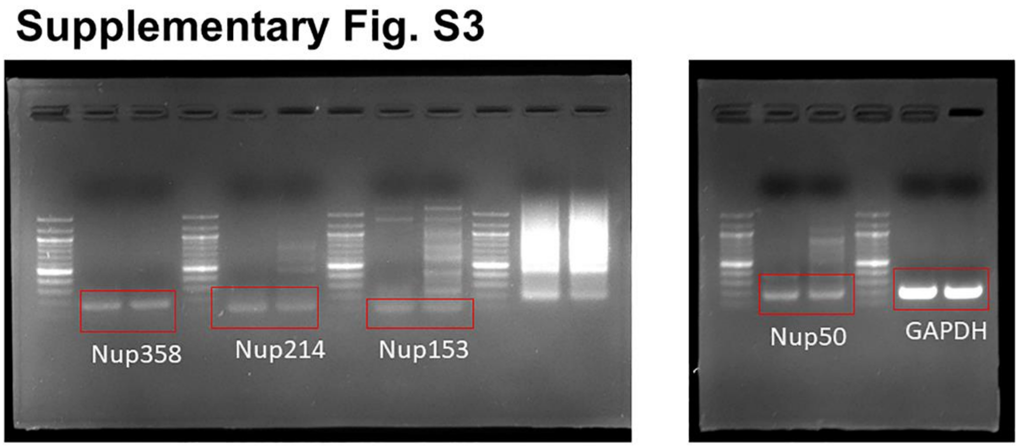

Supplementary Fig. S5

S5b

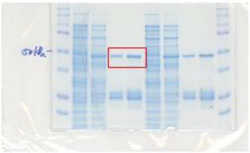

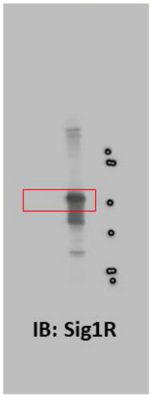

GST-hSig-1R

S5c

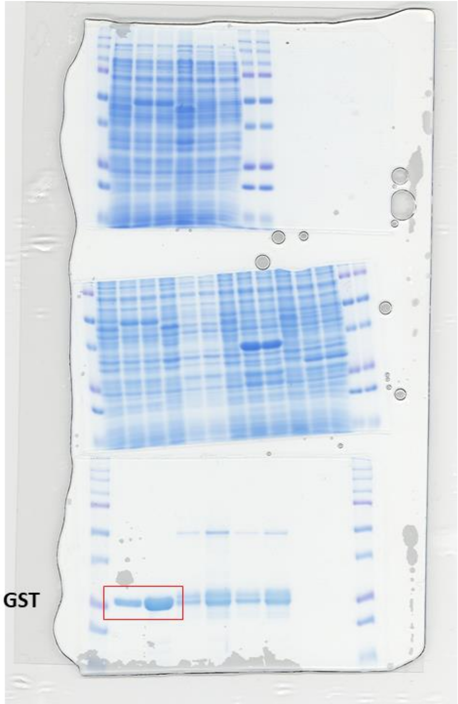

GST
